# Age, Dehydration, Respiratory Failure, Orientation Disturbance, and Blood Pressure Score Predicts In-hospital Mortality in HIV-negative Non-multidrug-resistant Smear-positive Pulmonary Tuberculosis in Japan

**DOI:** 10.1038/srep21610

**Published:** 2016-02-17

**Authors:** Kenjiro Nagai, Nobuyuki Horita, Takashi Sato, Masaki Yamamoto, Hideyuki Nagakura, Takeshi Kaneko

**Affiliations:** 1 Department of Pulmonology, Yokohama City University Graduate School of Medicine, Yokohama, Japan

## Abstract

The A-DROP scoring system was originally designed to assess clinical severity of community acquired pneumonia using the following parameters: advanced Age, Dehydration, Respiratory failure, Orientation disturbance (confusion); and, low blood Pressure. Total A-DROP score ranges zero to five assigning one point for each component, wherein five indicates the poorest prognosis. The purpose of this single-center retrospective study was to determine whether A-DROP could predict the risk for death in patients with pulmonary tuberculosis. We reviewed consecutive HIV-negative, non-multidrug-resistant smear-positive adult pulmonary tuberculosis patients. The cohort consisted of 134 men (38.8%), 211 women (61.2%), 272 who discharged alive (28.8%), and 73 who died in-hospital (21.2%) with a median age of 72 (IQR: 54–82) years. A one-point increase in the A-DROP score was associated with a higher risk for in-hospital mortality with odds ratio of 3.8 (95% confidence interval 2.8–5.2, P < 0.001). The area under receiver operating characteristics curve was 0.86. The total score cutoff of 1.5 provided the best Youden Index of 0.61. Using this criteria, total score >1.5, sensitivity was 85% and specificity was 76%. Kaplan-Meier curve clearly indicated that in-hospital mortality increased with higher A-DROP scores (Log-rank test <0.001). In conclusion, A-DROP score clearly indicate pulmonary tuberculosis in-hospital mortality.

Tuberculosis (TB), caused by *Mycobacterium tuberculosis*, is an infectious disease that has adversely affected human beings for many centuries. Although global TB incidence has fallen by an average of 1.5% per year since 2000 and is now 18% lower than the level of 2000, there are 9.6 million new TB cases and 1.5 million TB deaths a year^1^. Smear-positive TB patients usually need to be admitted to isolation wards. Many hospitalized TB patients cannot survive the long course of hospital treatment^2^,^3^. Some risk factors such as advanced age, chronic diseases, human immunodeficiency virus (HIV)- positive status, malnutrition, multidrug-resistant (MDR)-TB, impaired activities of daily living, and extensive infiltration as revealed by chest radiography lead to a poor life prognosis of patients with pulmonary TB^4^,^5^.

Pneumonia is another common pulmonary infectious disease that remains one of the major reasons for adult hospitalization and death, especially among the elderly population. For estimating the risk of death from community-acquired pneumonia (CAP), the Pneumonia Severity Index is the most solidly established scoring system^6^. The Pneumonia Severity Index consists of as many as 20 components and is too complicated for use in daily clinical practice; however, the Pneumonia Severity Index demonstrates how to evaluate patients with a potentially critical infectious disease. Another well-known scoring tool for pneumonia is CURB-65 (Confusion, Urea, Respiratory rate, Blood pressure, and Age >65)^7^. More recently, the Japanese Respiratory Society has proposed another 6-point scale (0–5) to assess the clinical severity of CAP; Age, Dehydration, Respiratory failure, Orientation disturbance, and blood Pressure (A-DROP). A-DROP is a modified version of CURB-65^8^,^9^.

CURB-65 was originally designed for CAP. However, we can also apply CURB-65 for other infectious diseases. A prospective study by Howell *et al.* revealed that CURB-65 is a useful tool to predict 28-day in-hospital mortality among patients who visit the emergency department^10^. Consequently, it is reasonable to consider that A-DROP might be applicable for infectious diseases other than CAP. The purpose of this retrospective study was to determine whether A-DROP could predict the risk of death in patients with pulmonary TB.

## Methods

This study was approved by the Institutional Review Board of Yokohama City University and was carried out in accordance with the approved protocol (Approved ID: B150701004).

### Patients

The study was a retrospective cohort study of in-patients admitted to Yokohama City University Hospital. The patient records were reviewed from April 2007 to May 2015 on the basis of the admission. All the consecutive patients admitted to the isolation wards with a primary diagnosis of pulmonary TB and satisfying the inclusion criteria were reviewed.

The inclusion criteria were as follows: (i) active smear-positive pulmonary TB diagnosed with sputum culture and smear, (ii) newly diagnosed disease (patients who had already started treatment in another hospital were excluded), (iii) age ≥15 years on admission, and (iv) admission after TB diagnosis.

Exclusion criteria were (i) HIV positive status, (ii) MDR-TB, i.e. resistance to both isoniazid and rifampicin, (iii) transferred out, i.e. patients moved to another facility before the negative infectivity was confirmed, (iv) treatment failure that was defined by the persistent smear positivity after 12 months of admission^11^.

If the patients were admitted twice, only the first admission was assessed.

#### A-DROP score

We evaluated the patients’ severity at admission using the A-DROP score^8^. All components of the A-DROP score were clearly described in the chart on admission.

The A-DROP scoring system assesses the following parameters: (i) Age (men ≥70 years, women ≥75 years); (ii) Dehydration (blood urea nitrogen ≥21 mg/dL); (iii) Respiratory failure (arterial oxygen saturation ≤90% or arterial oxygen pressure ≤60 mm Hg); (iv) Orientation disturbance (confusion); and (v) blood Pressure (systolic blood pressure ≤90 mmHg)^8^,^9^. One point was assigned to each of A-DROP components. The total score ranged 0–5, wherein five pointes suggested the poorest prognosis. Each component of A-DROP reflected that of CURB-65 after modification for use in Japan. The cutoffs for age were adapted for prolonged life expectancy in Japan. Given the prevalence of oxygen saturation monitors and blood gas analysis, the respiratory failure was assessed mechanically. The Adult CAP Guideline by the Japanese Respiratory Society recommended choosing a treatment setting as follows; ambulatory treatment for cases with a score of zero, ambulatory or hospital treatment for cases with scores of one and two, hospital treatment for cases with a score of three, intensive care unit treatment for cases with scores of four and five^9^. A-DROP and CURB-65 have similar prognostic ability for predicting mortality in CAP^8^

### Treatment, hospital course, and outcome

Patients were, for the first two months, treated with the standard daily combined therapy with isoniazid, rifampicin, pyrazinamide, and ethambutol (HRZE); or isoniazid, rifampicin, and ethambutol (HRE). Both treatments were followed by the combination therapy with isoniazid and rifampicin^12^. The HRZE regimen containing pyrazinamide was the principally preferred regimen, and the HRE regimen without pyrazinamide was mainly used for patients at high risk for drug-induced liver injury. The treatment choice was made by the physician who treated each patient. The dose of antibiotics were as follows: isoniazid 5 mg/kg/day (maximum 300 mg/day), rifampicin 10 mg/kg/day (maximum 600 mg/day), pyrazinamide 25 mg/kg/day (maximum 1500 mg/day), ethambutol 15–20 mg/kg/day (maximum 750–1000 mg/day)^12^. These doses generally followed the guidelines, however, the maximum doses for pyrazinamide and ethambutol were adjusted because the Japanese generally have small body weight. All patients were admitted to the isolation ward and antibiotics treatment under direct observation was initiated soon after admission.

The patients were discharged based on receiving effective therapy with the clinical improvement and after confirmation of negative infectivity^13^. The negative infectivity was confirmed when three or more consecutive sputum samples obtained on different days were smear-negative for acid-fast bacilli, or when appropriately taken sputum samples were culture negative three consecutive times^13^. Sputum was examined once a week.

The outcome of interest was in-hospital death due to any cause. Death certificates were used to determine the cause of death. People were classified as discharged alive if they were discharged from the hospital after smear-negativity was confirmed.

### Statistics

To compare patients who were discharged alive and patents who died in –hospital, Fisher’s exact test and a Mann-Whitney rank sum test were used for binary variables and continuous variables, respectively. The receiver operating characteristics (ROC) curve and area under the ROC curve (AUC) were used to evaluate the predictive ability of the A-DROP score for in-hospital death. A Kaplan-Meier curve and log-rank test were used to compare the survival of patients with each A-DROP score. For the Kaplan-Meier curve analysis, patients were censored when discharged alive. Univariate logistic regression analysis was used to estimate how a one-point increase in the total A-DROP score affected in-hospital mortality. Besides our primary goal evaluating how total A-DROP score could predict mortality, we additionally assessed whether each A-DROP component could predict mortality. Multiple logistic regression analysis was used to estimate how each component of the A-DROP score affected in-hospital mortality. The Cox proportional hazard model was used to calculate the hazard ratio (HR). Medians are presented with the interquartile range (IQR).

All analyses were performed using GraphPad Prism version 6.0 (GraphPad Software, San Diego, CA, USA) and Excel Toukei 2012 (SSRI, Tokyo, Japan).

## Results

### Patients

During the observation period, 366 patients satisfied the inclusion criteria. Among them, 20 were excluded after being transferred out and one was excluded due to having HIV positive status. None was excluded for MDR-TB or for treatment failure. Eventually, our cohort included 345 smear-positive HIV-negative non-MDR-TB patients (Table 1). The cohort consisted of 134 men (38.8%) and 211 women (61.2%) with a median age of 72 (IQR: 54–82) years. The most common co-morbidity was diabetes, from which 88 patients (25.5%) in the cohort suffered. On radiographic analysis, 144 patients (41.7%) had one or more pulmonary cavities and 253 (73.3%) patients had bilateral infiltration (Table 1).

The prevalence of A-DROP components at admission was as follows; age, 173 (50.1%); dehydration, 111 (32.2%); respiratory failure, 77 (22.3%); orientation disturbance, 54 (15.7%); and low blood pressure, 21 (6.1%) (Table 2). The median A-DROP score was 1 (IQR: 0–2). Among 345 patients, 117 (33.9%), 102 (29.6%), 64 (18.6%), 46 (13.3%), 12 (3.5%), and 4 (1.2%) patients had A-DROP scores of 0, 1, 2, 3, 4, and 5, respectively. The median total A-DROP score was 1 (IQR: 0–2). The average A-DROP score was 1.3 (standard deviation 1.2).

Concerning the chemotherapy regimen, 200 (58.0%) patients were treated with the HRZE regimen, 114 (33.0%) were treated with the HRE regimen, and 31 (9.0%) were treated with a non-standard regimen (Table 1). After infective negativity was confirmed, 272 (78.8%) patients were discharged alive. Meanwhile, 73 (21.2%) died in hospital. The causes of death were as follows: 61 due to TB, two due to hepatocellular carcinoma, two due to ischemic heart disease, one due to each of pneumonia, lung cancer, cerebral bleeding, cerebral infarction, liver cirrhosis, sepsis, prostate carcinoma, and congestive heart failure.

### Prognosis of patients and A-DROP score

Compared to the discharged-alive cases, a significantly higher proportion of died in-hospital cases had each component of the A-DROP; age, dehydration, respiratory failure, orientation disturbance, and low blood pressure were significantly associated with in-hospital death with odds ratios of 5.84, 6.37, 10.99, 7.21, and 3.05, respectively, with significance (Table 2). The median total A-DROP scores were 1 (IQR: 0–1) for discharged alive cases and 3 (IQR: 2–3) for died in-hospital cases.

**Figure 1:**
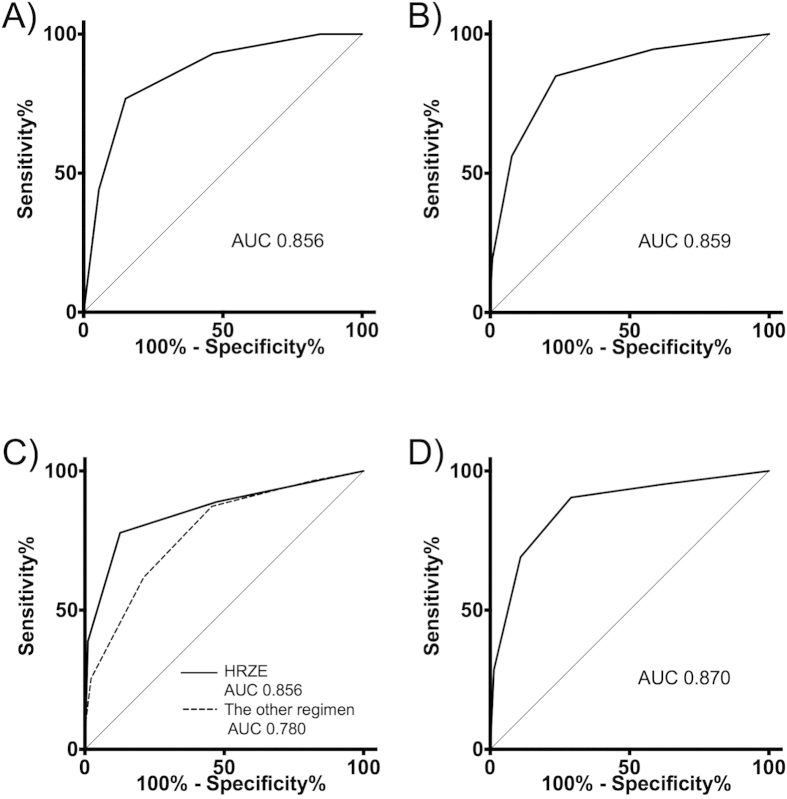
Receiver operating characteristic curves. (**A**) All-case analysis by total A-DROP score for in-hospital death. (**B**) All-case analysis by scoring excluding the low blood pressure for in-hospital death. (**C**) Subgroup analysis based on treatment regimen by total A-DROP score for in-hospital death. (**D**) All-case analysis by total A-DROP score for 28-day death.

In-hospital mortality was 3%, 7%, 33%, 59%, 83%, and 100% for patients who had an A-DROP score of 0, 1, 2, 3, 4, and 5, respectively (Table 2). The univariate logistic regression indicated that a one-point increase in the A-DROP score was associated with a higher risk for in-hospital mortality with an odds ratio of 3.83 (95% confidence interval (95%CI) 2.8–5.2, *p* < 0.001). An AUC of 0.856 suggests the A-DROP scoring system has good predictive ability for in-hospital mortality (Fig. 1A). The total score cutoff of 1.5 provided the best Youden Index of 0.61. Using this cutoff, sensitivity was 85% and specificity was 76% (Table 3, Fig. 1A). The Cox model indicated that the HR of a one-point increase of A-DROP score for in-hospital death was 2.44 (95%CI 2.06–2.90, *p* < 0.001).

**Figure 2:**
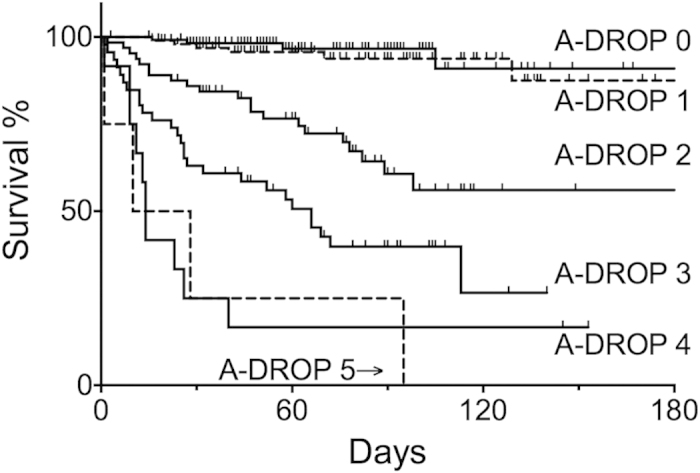
Kaplan-Meier curve for all-cause in-hospital death classified according to the total A-DROP score. Log-rank test: *p* < 0.001. The observation was not censored at 180^th^ day. However, we presented the data until the 180^th^ day because very limited number of cases were observed beyond the 180^th^ day.

The Kaplan-Meier curve clearly indicated that in-hospital mortality increased with higher A-DROP scores (Log-rank test < 0.001) (Fig. 2)

### Sensitivity analysis

Even though all the A-DROP components were associated with in-hospital mortality in the univariate analysis (Table 2), the multiple logistic regression and COX model suggested that the blood pressure component had little impact on in-hospital mortality (Table 4). After eliminating the blood pressure component, the AUC by the modified scoring (Age, Dehydration, Respiratory failure, Orientation disturbance) for in-hospital death was still as high as 0.859 (Fig. 1B). The Cox model suggested that the HR of one point increase of the modified score was 3.02 (95%CI 2.42–3.78, *p* < 0.001).

We divided our cohort into the HRZE treated subgroup (n = 200) and the other regimens subgroup (n = 145). The AUC for in-hospital death was 0.856 for the HRZE subgroup and 0.780 for the other regimen subgroup (Fig. 1C). Using the Cox model, the HR of a one-point increase of the A-DROP score for in-hospital death was 4.89 (95%CI 2.84–8.41, P < 0.001) and 1.98 (95%CI 1.59–2.46, *p* < 0.001) among the HRZE subgroup and the other regimens’ subgroup, respectively.

As a sensitivity analysis, we used 28-day death instead of in-hospital death. The AUC by A-DROP total score for 28-day death was 0.870 (Fig. 1D). The Cox model indicated the HR of a one-point increase of A-DROP score for 28-day death was 2.73 (95%CI 2.19–3.41, *p* < 0.001).

**Figure 3:**
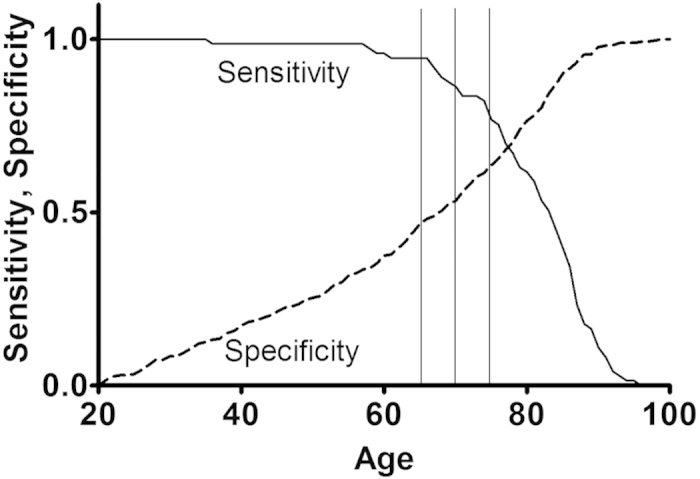
Sensitivity/specificity versus age plot. Sensitivity and specificity are for in-hospital death. A-DROP used cutoff age of 70 for men and 75 for women. CURB-65 used cutoff age of 65.

Because the A-DROP and CURB-65 use different cutoffs for age, we made a sensitivity/specificity versus age plot (Fig. 3). This showed that the cutoff age used for A-DROP had higher specificity and lower sensitivity than that used for CURB-65.

## Discussion

We retrospectively revealed that the A-DROP score effectively determines all-cause in-hospital mortality in HIV-negative non-MDR smear-positive TB. Sensitivity analyses based on treatment regimen and different outcome, i.e. in-hospital death and 28-day death, ensured the robustness of the result (Fig. 1). To our knowledge, this is the first study that has evaluated the prognostic ability of the A-DROP score for TB cases while previous studies evaluated CAP patients. The study results showed that each component of the A-DROP score, except for low blood pressure, along with the total A-DROP score had good predictive ability for in-hospital mortality for TB patients. We think the A-DROP score is useful when physicians treat TB patients for some purposes. First, the scoring system let us know which patients needed close monitoring during their hospital stay. Second, knowing the poor prognosis, patients, family, and physicians can discuss advanced directives concerning the intensive treatment. Some patients, especially the elderly, do not like to have intensive care such as mechanical ventilation^14^,^15^. Third, having an accurate prognosis of patients enables physicians to provide accurate information to patients. Provision of sufficient information is particularly important for admitted smear-positive TB patients because they usually have anxiety about the long-term hospitalization^16^.

The AUC by A-DROP score for death due to pneumonia has been repeatedly evaluated. According to an observational study with 2308 in-patients and out-patients by Jones *et al.*, the AUC according to an A-DROP score ≥2 for death due to pneumonia was 0.78^17^. Ishibashi *et al.* recently reported a retrospective study using data on 97 home care-based patients diagnosed with pneumonia^18^. In this study, the AUC by A-DROP score for in-hospital death was 0.78. Based on Ugajin’s retrospective cohort study, the AUC by A-DROP was 0.73 for death due to community-acquired pneumonia and 0.70 for nursing home-acquired pneumonia^19^. In this study, the AUC might have been slightly underestimated because the study included only patients aged ≥65 years. Another retrospective study by Ugajin *et al.* focusing on hospitalized CAP, AUC by A-DROP for death was 0.80^20^. Kasamatsu *et al.* reported a prospective study that was conducted in two Japanese hospitals with 226 hospitalized CAP patients. According to this study, the AUC by A-DROP for death was 0.88^21^. Shindo *et al.* retrospectively reported that the AUC by A-DROP for death in admitted patients with CAP was 0.85^8^. Overall, the AUC by A-DROP for death due to pneumonia was in the range 0.70–0.88. This suggests that the A-DROP score has good prognostic ability for pneumonia.

In the current study, AUC by A-DROP for in-hospital death for TB cases was as high as 0.856. This suggests that the A-DROP score has good prognostic ability for both lower respiratory infections, CAP and pulmonary TB. Given that the A-DROP was first developed for CAP, not for TB, it is surprising that the A-DROP has similar predictive ability for TB and CAP patients. The prognosis of CAP patients is affected by many factors besides the components of the A-DROP score, such as the types of causal bacteria and the choices of antibiotics. On the other hand, pulmonary TB is a homogeneous disease that is always caused by *Mycobacterium tuberculosis* and is usually treated with the same combined antibiotics regimen. Furthermore, we included only HIV-negative, non-MDR, smear-positive TB patients. Therefore, the prognoses of TB cases in the current study were less affected by factors other than A-DROP components.

One interesting finding is that blood pressure had little to do with the in-hospital mortality in the multiple-variate analysis (Table 4). In the CAP, especially pneumococcal pneumonia, with which patients may become sick in hours, low blood pressure may suggest septic shock due to severe infection. However, patients who suffer from TB usually become ill more gradually. Therefore, septic shock is not so common for TB patients. Here, we would like to discuss why the other four components had an impact on TB patients’ mortality. Age is a strong predictor of death in nearly all situations. Poor prognosis among the elderly has multiple explanations, such as multiple comorbidities, functional impairment, breakdown of the lungs’ epithelial barriers, changes in cellular and humoral immunity in specific cell populations, patient delay due to the poor perception of disease, and doctor delay due to atypical lung TB symptoms^22^. The physiological definition of dehydration is a decline in total body water volume compared to the euvolaemic state. Dehydration is a critical situation for human beings, especially for infants and the elderly, whose homeostasis does not function as well as that of young adults. For the elderly, dehydration can lead to an increase in falls, delirium, kidney failure, abnormal electrolytes, and deterioration of the immune system^23^. Respiratory failure suggests broad TB invasion of the lung, the large cavity of the lung, and possible co-morbid lung and heart diseases, all of which increase the risk of death.

The present study has a few limitations. First, this was designed as a retrospective study. However, all the data concerning A-DROP components and outcomes could be objectively extracted. Thus, the retrospective study design did not greatly affect the result. Second, we did not evaluate TB patients with HIV co-infection and MDR-TB. Thus, the external validity for different types of tuberculosis in a non-Japanese setting is not clear. The African and South-East Asia regions feature a high prevalence of HIV co-infection and high mortality rates. MDR-TB and retreatment cases are most often observed in the European region^24^. A different predictive rule is necessary for TB patients in these regions. Third, the reliability of A-DROP, especially the orientation component, had not been sufficiently evaluated in any previous study and could not be evaluated in the current study. Fourth, only 16 (4.6%) of our cases had A-DROP score of four or higher. Therefore, we can apply these high scores for only a limited number of cases.

Fifth, although Cox model is preferred to compare survival in two groups when we have time to event data, we have only “biased” time to event data for our cohort. Patients with lower A-DROP score (mild cases) got smear negativity after shorter duration of treatment. Therefore, patients with lower A-DROP score were censored after shorter observation. Patients are at very low risk for death after discharge. These facts clearly introduced a bias to underestimate the impact of A-DROP score when using Cox model. To overcome this limitation, we additionally compared in-hospital mortality. As estimated, in-hospital mortality expressed with OR suggested stronger impact for mortality.

In conclusion, this retrospective study of HIV-negative, non-MDR, smear-positive TB patients in a Japanese hospital showed that the A-DROP score predicted in-hospital mortality due to TB with a high accuracy. The A-DROP score may be a useful tool for determining the prognosis of admitted HIV-negative, non-MDR, smear-positive TB patients.

## Additional Information

**How to cite this article**: Nagai, K. *et al.* Age, Dehydration, Respiratory Failure, Orientation Disturbance, and Blood Pressure Score Predicts In-hospital Mortality in HIV-negative Non-multidrug-resistant Smear-positive Pulmonary Tuberculosis in Japan. *Sci. Rep.* **6**, 21610; doi: 10.1038/srep21610 (2016).

## Figures and Tables

**Table 1:** Baseline patient characteristics and treatment regimens.

	Total (N = 345)	Discharged alive (N = 272)	Died in-hospital (N = 73)	*p* value
Age (years)	72 (54–82)	67 (49–79)	83 (75.5–86)	<0.001
Sex (female)	134 (38.8%)	101 (37.1%)	33 (45.2%)	0.225
Cavity on X-ray	144 (41.7%)	116 (42.6%)	28 (38.4%)	0.593
Bilateral infiltration on X-ray	253 (73.3%)	189 (69.5%)	64 (87.7%)	0.002
Previous history of TB treatment	38 (11.0%)	29 (10.7%)	9 (12.3%)	0.676
Extra-pulmonary pulmonary TB	33 (9.6%)	27 (9.9%)	6 (8.2%)	0.824
Diabetes	88 (25.5%)	69 (25.4%)	19 (26.0%)	0.881
Immunosuppression	42 (12.2%)	27 (9.9%)	15 (20.5%)	0.025
Chronic cardiac disease	49 (14.2%)	30 (11.0%)	19 (26.0%)	0.002
Chronic pulmonary disease	41 (11.9%)	29 (10.7%)	12 (16.4%)	0.220
Chronic liver disease	40 (11.6%)	23 (8.5%)	17 (23.3%)	0.002
Chronic renal disease	42 (12.2%)	28 (10.3%)	14 (19.2%)	0.045
Active malignancy	41 (11.9%)	26 (9.6%)	15 (20.5%)	<0.001
Hemoglobin (g/dL)	11 (9.6–12.7)	11.4 (10.0–13.1)	9.9 (8.6–11.2)	<0.001
Aspartate aminotransferase (IU/dL)	26 (19–44)	24 (18–38)	39 (25–76.5)	<0.001
Alanine aminotransferase (IU/dL)	18 (12–32)	17.5 (12–30.75)	21 (14–41.5)	0.082
Blood urea nitrogen (mg/dL)	15 (12.0–23.0)	14 (11.0–19.8)	23 (16.5–33.0)	<0.001
Creatinine (mg/dL)	0.65 (0.51–0.90)	0.65 (0.53–0.88)	0.67 (0.48–1.12)	0.501
Treatment regimen				<0.001
HRZE	200 (58.0%)	182 (66.9%)	18 (24.7%)	
HRE	114 (33.0%)	74 (27.2%)	40 (54.8%)	
The other regimens	31 (9.0%)	16 (5.9%)	15 (20.5%)	

Continuous variables were presented as median (interquartile range).

%: proportion to numbers of total patients (N = 345), patients discharged alive (N = 272), or patients died in-hospital (N = 73).

HRZE: isoniazid + rifampicin + pyrazinamide + ethambutol. HRE: isoniazid + rifampicin + ethambutol.

*p* value: Fisher’s exact test or Mann-Whitney rank sum test was used for binary variables and ontinuous variables, respectively. Treatment regimens were compared using chi-square test.

**Table 2:** Comparison of A-DROP components and total score between discharged alive and died in-hospital cases.

	Total N = 345	Discharged alive N = 272	Died in-hospital N = 73	Odds ratio	*p* value
*Components*					
Age: >70 (men), >75 (women)	173 (50.1%)	114 (41.9%)	59 (80.8%)	5.84	<0.001
Dehydration	111 (32.2%)	63 (23.2%)	48 (65.8%)	6.37	<0.001
Respiratory failure	77 (22.3%)	33 (12.1%)	44 (60.3%)	10.99	<0.001
Orientation disturbance	54 (15.7%)	24 (8.8%)	30 (41.1%)	7.21	<0.001
blood Pressure	21 (6.1%)	12 (4.4%)	9 (12.3%)	3.05	0.023
*Total score*				In-hospital mortality	
0	117 (33.9%)	113 (41.5%)	4 (5.5%)	3%	<0.001
1	102 (29.6%)	95 (34.9%)	7 (9.6%)	7%	
2	64 (18.6%)	43 (15.8%)	21 (28.8%)	33%	
3	46 (13.3%)	19 (6.9%)	27 (37.0%)	59%	
4	12 (3.5%)	2 (0.7%)	10 (13.7%)	83%	
5	4 (1.2%)	0 (0.0%)	4 (5.5%)	100%	

Odds ratio was calculated in univarite manner.

*p* value: Fisher’s exact test or Mann-Whitney rank sum test was used for A-DROP components and total score, respectively.

**Table 3:** Prognostic ability of A-DROP score for in-hospital death of the admitted tuberculosis patients.

Cutoff	Sensitivity	Specificity	PPV	NPV
0/1	0.95	0.42	0.30	0.97
1/2	0.85	0.76	0.49	0.95
2/3	0.56	0.92	0.66	0.89
3/4	0.19	0.99	0.88	0.82
4/5	0.05	1.00	1.00	0.80

PPV: Positive predictive value. NPV: Negative predictive value.

**Table 4:** Results from multiple-variate analysis for in-hospital death.

Multiple logistic regression	Odds ratio	95%CI	*p* value
Age: >70 (men), >75 (women)	3.50	1.66–7.37	0.001
Dehydration	3.98	2.08–7.62	<0.001
Respiratory failure	5.97	3.05–11.68	<0.001
Orientation disturbance	3.36	1.60–7.08	0.001
blood Pressure	1.22	0.34–4.44	0.758
Multiple-variate Cox model	Hazard ratio		
Age: >70 (men), >75 (women)	2.22	1.22–4.02	0.009
Dehydration	3.77	2.29–6.20	<0.001
Respiratory failure	3.82	2.32–6.29	<0.001
Orientation disturbance	2.65	1.62–4.34	<0.001
blood Pressure	0.82	0.39–1.71	0.593
